# Supplementary material for: Causal Relationship between Aging and Anorexia Nervosa: A White-Matter-Microstructure-Mediated Mendelian Randomization Analysis
Source: Biomedicines. 2024 Aug 16;12(8):1874. doi: 10.3390/biomedicines12081874 (PMC11351342; doi:10.3390/biomedicines12081874)
Supplement: Supplementary file 1 [file biomedicines-12-01874-s001.zip › Supplementary Figure/Figure S8/Leaveoneout plot of MR analysis for TL to ubm-b-1980.pdf]

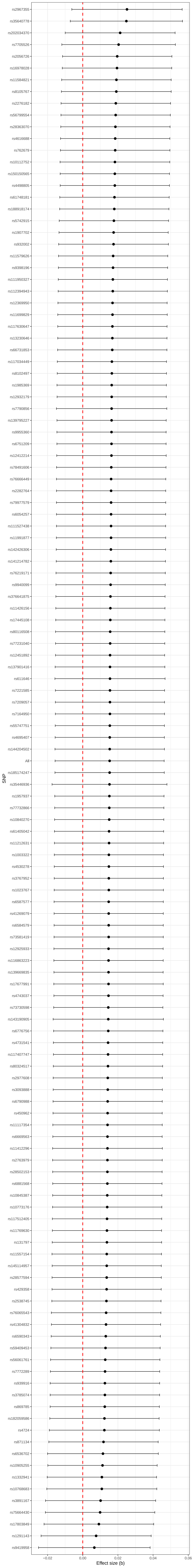
rs2967355
rs35640778
rs202034370
rs7705526
rs2056726
rs16978028
rs11584821
rs8105767
rs2276182
rs56799554
rs28363070
rs4616688
rs762679
rs10112752
rs150150565
rs4498805
rs61748181
rs188918174
rs5742915
rs1907702
rs932002
rs11579626
rs9398196
rs111950327
rs112394943
rs12369950
rs11699829
rs117630647
rs13230646
rs66731853
rs117034449
rs8102497
rs1985369
rs12932179
rs7790856
rs139795227
rs9955360
rs6751209
rs12412214
rs78491606
rs76666449
rs2282764
rs79977579
rs6054257
rs111527438
rs11991877
rs142426306
rs141214782
rs76219171
rs9940099
rs376641875
rs11426156
rs17445108
rs80116508
rs77231040
rs12451892
rs137901416
rs611646
rs7221585
rs7209057
rs7164950
rs55747751
rs4695407
rs144204502
All
rs185174247
rs35446936
rs1957937
rs77732866
rs10840270
rs61405042
rs11212631
rs1003322
rs4530278
rs3767952
rs1023767
rs6587577
rs41269079
rs6584579
rs73581419
rs12925933
rs116863223
rs139669835
rs17677991
rs4743037
rs73730598
rs143190905
rs6776756
rs4731541
rs117407747
rs80324517
rs2977608
rs3093888
rs6790988
rs450962
rs11117354
rs6669563
rs11412296
rs2763979
rs28502153
rs6881568
rs10845387
rs10773176
rs117512405
rs11769630
rs131797
rs11557154
rs145114957
rs28577594
rs429358
rs2538745
rs76065543
rs41304832
rs6590343
rs59409453
rs56061761
rs7772289
rs939916
rs3785074
rs869785
rs182059586
rs4724
rs871134
rs6536702
rs10905255
rs1332941
rs10768683
rs3891167
rs75664430
rs17803849
rs1291143
rs9419958
SNP
−0.02
0.00
0.02
0.04
0.06
Effect size (b)
